# Supplementary material for: Generalized FitzHugh-Nagumo equations with Caputo gH-differentiability: A novel fuzzy fractional approach to digital memristor networks
Source: PLoS One. 2026 Feb 3;21(2):e0339866. doi: 10.1371/journal.pone.0339866 (PMC12867333; doi:10.1371/journal.pone.0339866)
Supplement: Attachment — Submitted filename: Reviewer response of PONE-D-25-28173.pdf [file pone.0339866.s001.pdf]

August 25, 2025
Journal: PLOS One
Manuscript ID: Ms. Ref. No.: PONE-D-25-28173
Title: Generalized FitzHugh-Nagumo equations with Caputo gH-differentiability: A novel fuzzy fractional approach to digital Memristor networks

Dear Emily Chenette,

We sincerely appreciate the opportunity to revise our manuscript for possible publication in PLOS One and would like to thank you and the reviewers for their guidance and comments in your letter dated August 25, 2025. We have carefully incorporated their suggestions and made the necessary changes, including fixing grammatical and typing errors and improving the writing in certain areas. We are grateful for the valuable input provided by the reviewers and agree with all the comments made. We hope that the revised version of our paper meets their expectations and is suitable for publication in your esteemed journal.

Thank you for your time and consideration.

Best Regards,
Dr. G. Muhammad
(Corresponding Author)

Response to the Comments of Reviewer 1

1.Explain clearly fuzzy fractional generalized FitzHugh-Nagumo differential equations ?

Our Response.

We appreciate this constructive comment. We have revised and clearly explained the fuzzy fractional generalized FitzHugh-Nagumo differential equations in section 3 according to your suggestions.

2. How do fuzzy FFHNDEs enhance the modeling of neurodynamical systems by incorporating uncertainty, memory effects, and nonlinear behavior?

Our Response.

We appreciate this constructive comment. We have explained that how fuzzy fractional generalized FitzHugh-Nagumo differential equations enhance the modeling of neurodynamical systems by incorporating uncertainty, memory effects, and nonlinear behavior in introduction and section 5.

Response to the Comments of Reviewer 2

1. This manuscript lacks the validation of analytical solutions. Author must provide the evidence that solution are accurate and relevant to real-world memristor network. I suggest (a)..., (b)...

Our Response. We appreciate your constructive comment. We have provided the detailed comparison of the proposed generalized fuzzy solutions and provided the detailed discussion in order to validate our results in section 4.

2. Figures could be improved. Add axis labels and legends to make more easier to understand.

Our Response. Thank you so much for your valuable comment. We have improved the figures by showing the axes clearly.

3. The English language needs significant improvement. There are numerous grammatical errors, inconsistencies in notation.

Our Response.

Thank you so much for your valuable comment. We have corrected the grammatical errors and improved the manuscript by adding concluding sentences to each section.

Response to the Comments of Reviewer 3

1. The physical motivation for applying the Caputo gH-differentiable fractional framework to the FHN model should be explained more clearly, especially its specific advantage over other fractional definitions in the context of memristor network dynamics.

Our Response.

Thank you so much for your valuable comment. We have provided the physical motivation for applying the Caputo gH-differentiable in the introduction section and explained the proposed model in section 3 and the real-world benefits of the propsed model as memristors networks in section 5.

2,3. The derivation of the fuzzy fractional model lacks intermediate mathematical steps in some areasadding these would improve transparency and reproducibility.

Our Response.

Thank you so much for your valuable comment. We have revised the derivation of the fuzzy fractional model and organized the fuzzy solutions in a arranged way in section 3.

4. The link between the fuzzy fractional formulation and the memristor network model should be strengthened by explaining the mapping of FHN variables to memristor parameters.

Our Response.

Thank you so much for your valuable comment. We have provided the one-one correspondence of proposed model as an application of fuzzy memristor networks in in section 5.

5,6,7. Numerical implementation details (e.g., discretization method, time step size, stability considerations) are too brief these should be expanded for clarity. A brief comparative discussion with other fractional-order modeling techniques (e.g., Atangana-Baleanu, Caputo-Fabrizio derivatives) could help highlight the novelty of the chosen method.

Our Response.

Thank you so much for your valuable comment. We have provided the numerical solutions at fixed fuzzy parameters and variables in order to determine the novelty and accuracy of the solutions of proposed model. We have provided the comparative discussion of fuzzy solutions with the other existing techniques to show the novelty of the fuzzy solutions.

8. The conclusion should elaborate more on the potential practical implications in neuromorphic computing and whether the proposed approach is computationally feasible for large-scale memristor arrays.

Our Response.
We have revised the conclusion section and explained the results in a broader way.

9. For enhancing the introduction section with the new publications, old references may be replaced with new ones such as: Artificial neural network validation of MHD natural bioconvection in a square enclosure: entropic analysis and optimization

Our Response. We have revised the introduction section and added the new pubilcations related to the concerned work according to your suggestions.

Response to the Comments of Reviewer 4

The manuscript demonstrates significant potential and originality but requires substantial improvements in:1. Clearer articulation of novelty.2. Comparative and statistical validation of results.....

Our Response. Thank you very much for your constructive commets on this manuscript. We have revised the article and provided clear understanding of novelty, comparative analysis, stronger discussion, and updated references related to proposed model.

Response to the Comments of Reviewer 5

The paper addresses the fuzzy fractional generalized FitzHugh-Nagumo differential equations (FFGFH-NDEs), which are crucial in biological systems...

Our Response. Thank you very much for your constructive comments on this manuscript.We have provided the real-world application of the proposed model in memristors networks, added the numerical results of fuzzy solutions for fixed fuzzy parameters and variables, comparative analysis of fuzzy solutions with the exixting solutions and the graphical representations in the clear way according to the valuable suggestions.
Thank you very much!!
