# Supplementary material for: Generalized FitzHugh-Nagumo equations with Caputo gH-differentiability: A novel fuzzy fractional approach to digital memristor networks
Source: PLoS One. 2026 Feb 3;21(2):e0339866. doi: 10.1371/journal.pone.0339866 (PMC12867333; doi:10.1371/journal.pone.0339866)
Supplement: Attachment — Submitted filename: Reviewer response of PONE-D-25-28173R2.pdf [file pone.0339866.s002.pdf]

November 19, 2025
Journal: PLOS One
Manuscript ID: Ms. Ref. No.: PONE-D-25-28173
Title: Generalized FitzHugh-Nagumo equations with Caputo gH-differentiability: A novel fuzzy fractional approach to digital Memristor networks

Dear Emily Chenette,

We sincerely appreciate the opportunity to revise our manuscript for possible publication in PLOS One and would like to thank you and the reviewers for their guidance and comments in your letter dated November 19, 2025. We have carefully incorporated their suggestions and made the necessary changes, including fixing grammatical and typing errors and improving the writing in certain areas. We are grateful for the valuable input provided by the reviewers and agree with all the comments made. We hope that the revised version of our paper meets their expectations and is suitable for publication in your esteemed journal.

Thank you for your time and consideration.

Best Regards,
Dr. G. Muhammad
(Corresponding Author)

Response to the Comments of Reviewer 2

1. Summary Table of Differentiability Cases: Include a concise table summarizing all differentiability types and corresponding solution forms.

   Our Response.

   We appreciate this valuable comment. We have presented the summary table summarizing all differentiability types and corresponding solution forms.

2. Schematic Diagram: Add a visual diagram illustrating the mapping between FFGFH-NDEs components and memristor network elements.

   Our Response.

   We appreciate this worthy comment. We have presented the schematic diagrams illustrating the mapping between FFGFH-NDEs components and memristor network.

3. Notation Table: Include a table of symbols and notations used throughout the manuscript for clarity.

   Our Response. We appreciate your comment. We have presented the notation table of symbols and notation used throughout the manuscript.

4. Broader Implications: Expand the discussion on how the proposed model could be extended to other domains such as fuzzy control systems or biological signal processing.

   Our Response. Thank you so much for your constructive comment. We have presented the discussion on the extension of proposed model for suggested domains. We have also revised and checked the correctness of all the references.
